# Identification of the molecular mechanisms associated with acute type A aortic dissection through bioinformatics methods

**DOI:** 10.1590/1414-431X20198950

**Published:** 2019-11-07

**Authors:** Tao Jiang, Liangyi Si

**Affiliations:** Cardiovascular Department, The Third Affiliated Hospital of Chongqing Medical University, Chongqing, China

**Keywords:** Aortic dissection, Differentially expressed genes, Functional enrichment analysis, Protein-protein interaction network, microRNAs, Small molecules

## Abstract

Aortic dissection is characterized by the redirection of blood flow, which flows through an intimal tear into the aortic media. The purpose of this study was to find potential acute type A aortic dissection (AAAD)-related genes and molecular mechanisms by bioinformatics. The gene expression profiles of GSE52093 were obtained from Gene Expression Omnibus (GEO) database, including 7 AAAD samples and 5 normal samples. The differentially expressed genes (DEGs) were detected between AAAD and normal samples. The functional annotation and pathway enrichment analysis were conducted through the Database for Annotation, Visualization and Integration Discovery (DAVID). A protein-protein interaction network was established by the Search Tool for the Retrieval of Interacting Genes (STRING) software. The microRNAs (miRNAs) of these differentially expressed genes were predicted using <microRNA.org> database. Moreover, DEGs were analyzed in the comparative toxicogenomics (CTD) database to screen out the potential therapeutic small molecules. As a result, there were 172 DEGs identified in patients with AAAD. These DEGs were significantly enriched in 6 pathways, including cell cycle, oocyte meiosis, DNA replication, extracellular matrix-receptor interaction, and mineral absorption pathway. Notably, CDC20, CDK1, CHEK1, KIF20A, MCM10, PBK, PTTG1, RACGAP, and TOP2A were crucial genes with a high degree in the protein-protein interaction network. Furthermore, potential miRNAs (miR-301, miR-302 family, and miR-130 family) were identified. In addition, small molecules like azathioprine and zoledronic acid were identified to be potential drugs for AAAD.

## Introduction

Aortic dissection (AD) is the most common and destructive disease involving the aorta. Compared with the rupture of abdominal aortic aneurysms, its occurrence is 2 times higher in the United States (1). Based on the Stanford classification method, type A aortic dissections (AAD) involve the ascending aorta, while type B aortic dissections involve the descending aorta. Because of severe complications (aortic regurgitation, lethal malperfusion syndrome, cardiac failure, and stroke), the mortality rate of aortic dissection is still high. For the most severe form, acute type A aortic dissection (AAAD), the mortality rate reaches 26% in patients who underwent surgery, but up to 58% in patients treated noninvasively due to advanced age or complications (2).

Although various risk factors have been proven to damage the aortic wall and cause dissection, the mechanism of AD still remains unclear. Previous studies have indicated that genes and microRNAs (miRNAs) are involved in AD.

Various mutations in connective tissue genes are related to AD (3). FBN1 mutations lead to the progression of aortic aneurysms and dissections, as well as susceptibility to skeletal and ocular features (4). Patients carrying TGFB1 or TGFB2 mutation have a higher risk of suffering aneurysms and dissections in the aorta and other arteries (4). The median survival of patients with COL3A1 mutation is 48 years and most deaths are caused by thoracic or abdominal dissection (5,6). In addition, microRNAs may also play important roles in the pathogenesis of AD. Overexpression of miR-30a promotes the progression of AD, possibly by targeting lysyl oxidase (7). MiR-320 could downregulate the expression of MMPs by macrophages in AD patients (8). MiR-21 knockout aggravated AngII-induced thoracic aortic dissection formation in mice, which was related to the dysfunction of TGF-β signaling (9). MiR-134-5p could effectively inhibit phenotypic switch and migration of vascular smooth muscle cells (VSMCs) by targeting the STAT5B/ITGB1 pathway (10). The downregulation of the miR143/145 gene cluster promoted a phenotypic switch of VSMCs through the TGF-β1 signaling pathway (11).

Microarray analysis of gene expression by bioinformatics has been widely used to find crucial genes and biological processes in AAD. In this study, we reanalyzed gene expression profiles of GSE52093 (12) to find differentially expressed genes (DEGs) that may induce AAAD development. Then, functional annotation, pathway, protein-protein interaction (PPI), and potential miRNAs, as well as small molecules associated with AAAD, were analyzed by bioinformatics methods. These results may facilitate the understanding of underlying molecular mechanisms and the finding of potential drugs for AAAD.

## Material and Methods

### Dataset

The gene expression profiles of GSE52093 (12) were obtained from the Gene Expression Omnibus (GEO) database (http://www.ncbi.nlm.nih.gov/geo/). This dataset includes five normal ascending aorta samples from normal donors and seven dissected ascending aorta samples from patients with AAAD. The Illumina HumanHT-12 v4.0 expression beadchip (USA) was employed to analyze the samples.

### Data preprocessing and identification of DEGs

The raw data were normalized by the Geoquery package (version 2.40.0; http://www.bioconductor.org/packages/release/bioc/html/GEOquery.html) (13). After background correction, data normalization, and determination of expression levels, the gene expression between AAAD and normal samples was compared using the linear regression model and empirical Bayes moderated *t*-test. Then, the fold change (FC) of gene expression between AAAD and normal samples was obtained, and the false discovery rate (FDR) was calculated through the Benjamini and Hochberg procedure (14), which is one of the multiple testing correction techniques to control family-wide FDR under or equal to 0.05. DEGs were identified with a | log FC | ≥1 and an FDR<0.05.

### Function annotation and pathway enrichment analysis of DEGs

Gene ontology (GO) and pathway enrichment analysis of DEGs were conducted via the database of Annotation, Visualization and Integration Discovery (DAVID; version 6.8; http://david.abcc.ncifcrf.gov/) (15). The enrichment of each GO term and Kyoto Encyclopedia of Genes and Genomes (KEGG) pathway from the DEGs was tested one-by-one in a linear model and enrichment P value was calculated by Fisher's exact test and hypergeometric distribution. The Benjamini adjusted P value of <0.05 was set as the cut-off value for screening out significant GO terms (16) and the KEGG pathways (17).

### PPI network construction

A PPI network of DEGs was established by the Search Tool for the Retrieval of Interacting Genes (STRING; version 11.0; http://string-db.org/) (18). The combination score in STRING is an integration of different types of prediction evidence including text-mining, experiments, databases, co-expression, neighborhood, gene fusion, and co-occurrence (18). A lower score means more interaction, but also more false positives. Only interaction pairs with a combination score >0.4 were selected to construct a PPI network. The protein was represented by a node, and the interaction between paired proteins was represented by an undirected line. The score of each node was calculated, which related to the number of interactions between proteins. Then Hub genes were selected based on the score.

### Identification of the potential microRNAs and small molecules

MicroRNAs play crucial roles in AAAD. microRNA.org (http://www.microrna.org) (19) is a comprehensive database of target predictions and expression profiles for microRNAs. To identify potential microRNAs of the DEGs, enrichment analysis was conducted based on the microRNA.org database. microRNA target predictions are based on the mirSVR algorithm, which incorporates current biological knowledge on target rules and the use of an up-to-date compendium of mammalian microRNAs (19). The algorithm trains a regression model on the sequence and contextual features extracted from miRanda-predicted target sites. The FDR<0.05 was the cutoff value.

The comparative toxicogenomics database (CTD; http://ctdbase.org/) (20) provides manually curated information on chemical-gene/protein interactions using a hierarchical interaction-type vocabulary that characterizes common physical, regulatory, and biochemical interactions between chemicals and genes or proteins. In order to identify the small molecules associated with AAAD, the identified DEGs were analyzed in the CTD database. The criterion was set as FDR<0.05.

## Results

### Identification of DEGs

**Figure 1.**
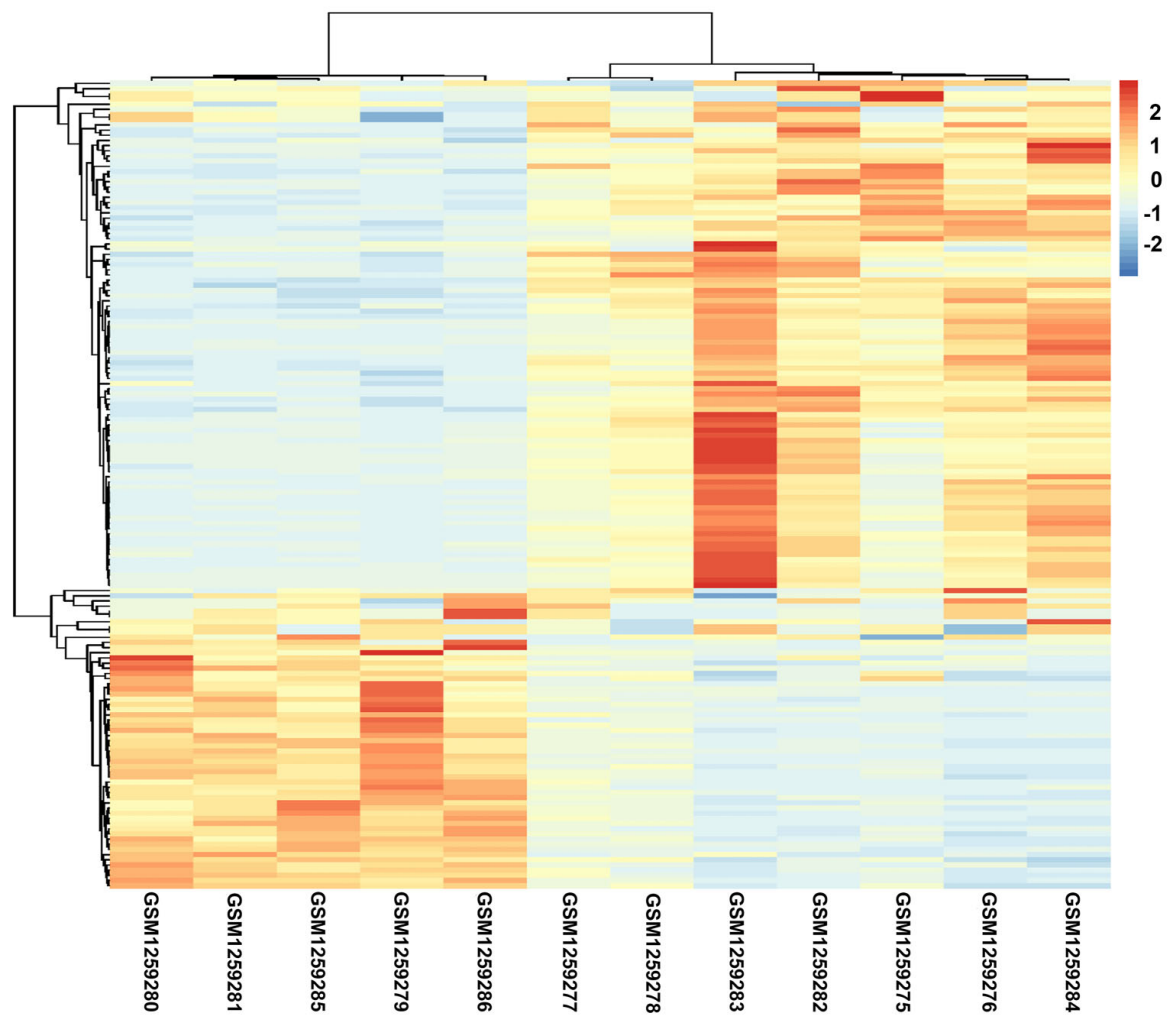
Hierarchical cluster analysis of the differentially expressed genes. GSMxxxxxxx: the accession number of a certain sample in the Gene Expression Omnibus database. GSM1259279–1259281, GSM1259285, and GSM1259286 represent normal samples, while GSM1259275–1259278 and GSM1259282–1259284 represent acute type A aortic dissection samples. The blue and red bars represent low and high expression levels, respectively.

Based on the cutoff of | log fold change (FC) | ≥1 and FDR<0.05, 172 DEGs were identified between the AAAD and normal samples, including 108 upregulated genes and 64 downregulated genes. For the identified DEGs, hierarchical cluster analysis was performed, and AAAD samples were obviously separated from normal samples, indicating the reliability of the DEGs (Figure 1).

### GO functional annotation and pathway enrichment analysis

**Figure 2.**
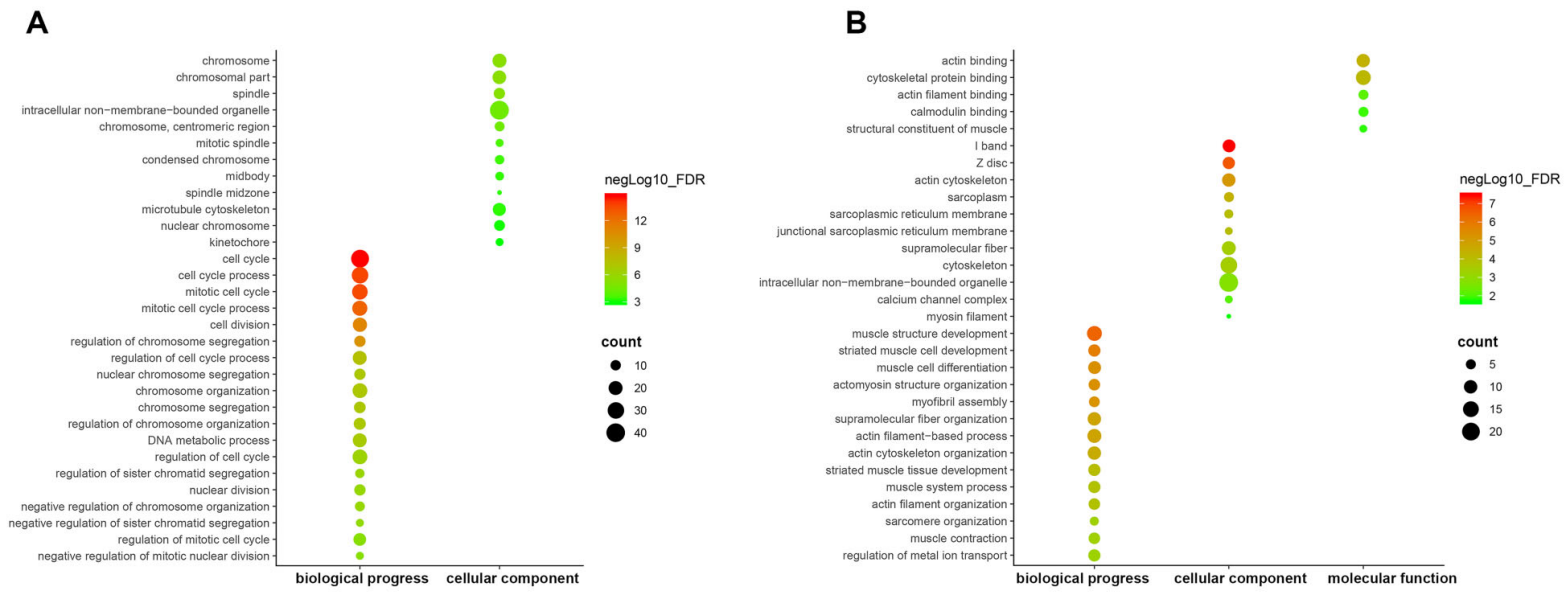
Gene Ontology (GO) functional annotation enrichment analysis of identified differentially expressed genes. X-axis represents GO terms, including biological progress, molecular function, and cellular component while Y-axis represents the names of GO terms. The green and red points represent high and low false discovery rate values, and the size of points indicates gene count. **A**, Enrichment result of upregulated genes; **B**, Enrichment result of downregulated genes.

The upregulated and downregulated genes were analyzed by the database of Annotation, Visualization and Integration Discovery. The upregulated genes were significantly enriched in several GO terms, including cell cycle, mitotic cell cycle, cell division, chromosome, and spindle, but these genes were not enriched in any molecular function. The downregulated genes were also enriched in several GO terms, such as muscle structure development, cytoskeletal protein binding, and I band (Figure 2).

Based on the KEGG pathway database, the upregulated genes were enriched in multiple signaling pathways, including cell cycle, oocyte meiosis, DNA replication, ECM-receptor interaction, and mineral absorption. In contrast, the downregulated genes were enriched in only one signaling pathway, circadian rhythm (Table 1).

**Table 1.** Kyoto Encyclopedia of Genes and Genomes (KEGG) pathway enrichment analysis of the differentially expressed genes (DEGs).

DEGs	KEGG Pathway	FDR	Gene count	Genes
Upregulated genes	hsa04110: Cell cycle	1.34E-06	9	BUB1, CDC20, CDC7, CHEK1, ESPL1, MAD2L2, MCM2, MCM4, PTTG1
	hsa04114: Oocyte meiosis	0.0133	5	BUB1, CDC20, ESPL1, MAD2L2, PTTG1
	hsa03030: DNA replication	0.0225	3	FEN1, MCM2, MCM4
	hsa04512: ECM-receptor interaction	0.0225	4	COL6A3, HMMR, ITGA2, SPP1
	hsa04978: Mineral absorption	0.0399	3	MT1E, MT2A, STEAP1
Downregulated genes	hsa04710: Circadian rhythm	0.0058	3	PER2, PER3, RORA

FDR: false discovery rate.

### Construction of PPI network and identification of hub genes

**Figure 3.**
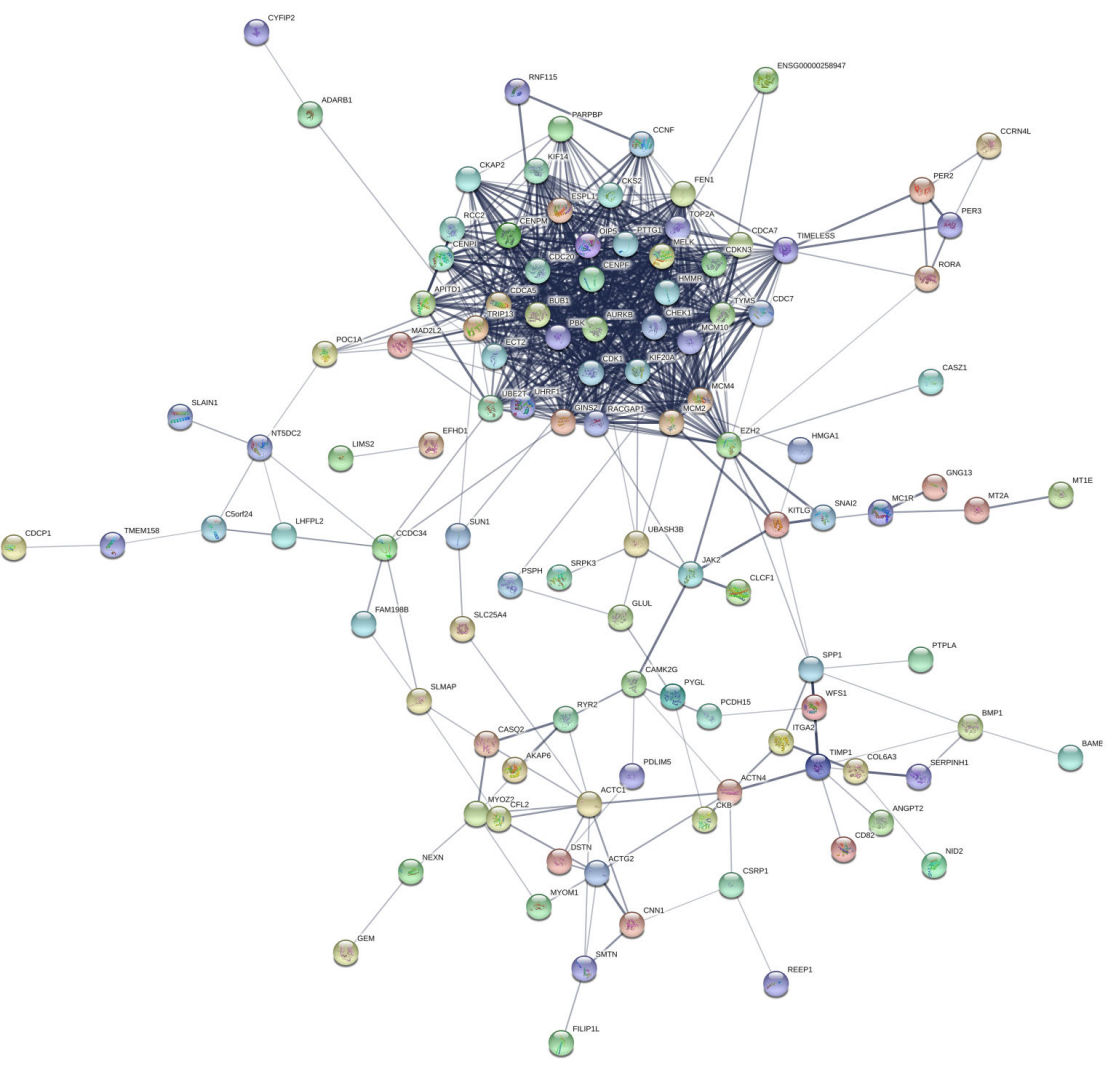
Differentially expressed genes in the protein-protein interaction network. The protein is represented by a node, and the interaction between paired proteins is represented by an undirected line.

The PPI network of 172 DEGs was established using the STRING database (Figure 3). After calculating the score of each gene, 9 DEGs with a degree of >20 were considered crucial for AAAD, including CDC20 (degree, 20.23), CDK1 (degree, 28.52), CHEK1 (degree, 22.54), KIF20A (degree, 23.50), MCM10 (degree, 23.83), PBK (degree, 28.91), PTTG1 (degree, 20.26), RACGAP (degree, 24.80), and TOP2A (degree, 29.14) (Table 2).

**Table 2.** Protein-protein interaction network degree and gene expression of identified hub genes.

Genes	Degree	Log FC	FDR
CDC20	20.23	4.15	0.0249
CDK1	28.52	2.85	0.0355
CHEK1	22.54	3.01	0.0276
KIF20A	23.50	5.31	0.0344
MCM10	23.83	3.22	0.0370
PBK	28.91	5.58	0.0344
PTTG1	20.26	2.77	0.0334
RACGAP	24.80	1.82	0.0443
TOP2A	29.14	4.43	0.0244

Log FC: log fold change; FDR: false discovery rate.

### Potential microRNAs and small molecules associated with AAAD

According to the microRNA.org database, the potential microRNAs of DEGs were screened out. The top 20 microRNAs, including miR-888, miR-4262, miR-301a, miR-301b, and miR-1264, are listed in Table 3. The miR-301 family, miR-302 family, and miR-130 family were among the most remarkable microRNAs, and most microRNAs targeted ANGPT2, REEP1, CFL2, and PBK.

**Table 3.** Top 20 microRNAs of the differentially expressed genes.

microRNA	FDR	Gene count	Target genes
miR-888	2.49E-14	25	TMEM30B, SPP1, NFKBIZ, FHOD3, ACTN4, etc.
miR-4262	3.52E-13	25	AKAP6, CDKN3, COL6A3, VCX3A, SLC25A4, etc.
miR-301b	1.97E-12	23	ANGPT2, POC1A, REEP1, CFL2, MYOZ2, etc.
miR-301a	2.02E-12	23	ANGPT2, POC1A, REEP1, CFL2, MYOZ2, etc.
miR-1264	4.50E-12	23	NID2, CAMK2G, PBK, MYOZ2, FHOD3, etc.
miR-130a	5.72E-12	21	AKAP6, POC1A, REEP1, CFL2, PBK, etc.
miR-499-5p	5.80E-12	20	TMEM30B, REEP1, CENPF, CFL2, GNG13, etc.
miR-130b	7.07E-12	21	AKAP6, POC1A, REEP1, CFL2, PBK, etc.
miR-2053	1.11E-11	24	AKAP6, NID2, DSTN, ANGPT2, CENPF, etc.
miR-141	1.51E-11	20	SLC25A4, MYOZ2, MAP3K7CL, MCM10, EZH2, etc.
miR-144	1.82E-11	24	AKAP6, CDKN3, TMEM30B, NID2, ANGPT2, etc.
miR-515-5p	2.19E-11	22	AKAP6, NID2, MYOZ2, NFKBIZ, MAP3K7CL, etc.
miR-429	4.27E-11	23	ANGPT2, REEP1, SLC25A4, CFL2, ACTC1, etc.
miR-520g	5.08E-11	20	ANGPT2, SPP1, PBK, FHOD3, MAP3K7CL, etc.
miR-200c	1.20E-10	22	ANGPT2, REEP1, SLC25A4, CFL2, ACTC1, etc.
miR-302a	1.21E-10	20	ANGPT2, POC1A, CFL2, PBK, MCM10, etc.
miR-302c	1.44E-10	20	NID2, POC1A, CFL2, PBK, CASQ2, etc.
miR-520h	1.57E-10	19	ANGPT2, SPP1, PBK, FHOD3, MAP3K7CL, etc.
miR-302b	2.00E-10	20	POC1A, CFL2, SUN1, PBK, CASQ2, etc.
miR-589	2.09E-10	22	CAMK2G, NFKBIZ, CHST1, CHEK1, HMMR, etc.

miR: microRNA; FDR: false discovery rate.

According to the CTD database, DEGs were analyzed to find potential small molecule drugs. Several small molecules were screened out to have significant correlations with the DEGs. The top 20 small molecules are listed in Table 4, such as palbociclib, dasatinib, azathioprine, and zoledronic acid. CDKN3, PTTG1, and PBK can be targeted by several small molecules.

**Table 4.** Top 20 small molecules of the differentially expressed genes.

Molecule in CTD	FDR	Gene count	Target gene
Palbociclib	9.49E-25	27	CDKN3, PTTG1, CENPF, PBK, OIP5, et al.
Dasatinib	1.52E-19	33	CDKN3, PTTG1, CENPF, PBK, ACTC1, et al.
Azathioprine	8.35E-19	36	CDKN3, NID2, CENPF, PBK, MCM2, et al.
2,3-bis(3′-hydroxybenzyl) butyrolactone	1.52E-18	42	THSD4, CDKN3, PTTG1, CENPF, PBK, et al.
Troglitazone	1.21E-16	46	CDKN3, NID2, PTTG1, ANGPT2, CENPF, et al.
Vinylidene chloride	1.83E-16	48	ENTPD7, CDKN3, NID2, PHLDA1, et al.
Zoledronic acid	4.37E-15	46	CDKN3, NID2, CENPF, PBK, ACTG2, et al.
Trimellitic anhydride	4.78E-15	52	CDKN3, PTTG1, POC1A, SLC25A4, PBK, et al.
Amphotericin B, deoxycholate drug combination	6.20E-15	26	CENPF, SPP1, MCM2, NFKBIZ, MCM4, et al.
Fluorouracil	6.33E-15	44	CDKN3, COL6A3, PTTG1, ANGPT2, ACTC1, et al.
Coumestrol	7.33E-15	50	THSD4, CDKN3, PTTG1, POC1A, CENPF, et al.
Lucanthone	9.66E-13	19	CDKN3, CENPF, PBK, HMMR, MCM10, et al.
1,4-bis(2-(3,5- dichloropyridyloxy)) benzene	7.68E-12	44	CDKN3, POC1A, REEP1, PHLDA1, CENPF, et al.
Mustard Gas	1.78E-11	39	CDKN3, PHLDA1, SPP1, PBK, MCM2, et al.
Polychlorinated Biphenyls	4.97E-11	27	CDKN3, CENPF, SPP1, PBK, MCM2, et al.
Ozone	1.56E-10	37	CDKN3, MYOM1, PHLDA1, CENPF, SPP1, et al.
2,4,5,2′,4′,5′- hexachlorobiphenyl	1.64E-10	44	COL6A3, PTTG1, CENPF, SPP1, TMEM158, et al.
benzo(b)fluoranthene	3.46E-10	40	THSD4, CDKN3, TMEM30B, PTTG1, REEP1, et al.
propionaldehyde	4.91E-10	45	NID2, PTTG1, POC1A, PHLDA1, CENPF, et al.
2,4,4′-trichlorobiphenyl	5.09E-10	38	COL6A3, PTTG1, CENPF, GNG13, PBK, et al.

CTD: comparative toxicogenomics database; FDR: false discovery rate.

## Discussion

AD is characterized by the redirection of blood flow, which flows through an intimal tear into the aortic media. It is the most common fatal disease of the aorta. Although many studies have been carried out to explore the pathogenesis of aortic dissection, the mechanism of aortic dissection development and progression remains unclear. In this study, we identified DEGs between normal and AAAD samples, conducted function annotation and pathway enrichment analysis of DEGs, and predicted potential miRNAs and small molecules associated with AAAD.

Microarray analysis of gene expression based on bioinformatics has been widely used to find AD-related genes. According to the gene expression dataset GSE52093, Pan et al. (12) found 2737 differentially expressed genes with the criteria of FDR<0.05. However, we screened out 172 DEGs with the criteria of | log fold change (FC) | ≥1 and FDR<0.05. The differences in the number of DEGs between our study and the previous study might be due to different analysis criteria; however, our criteria are the most commonly used for DEG screening.

Additionally, the previous study had found that the cell cycle pathway was involved in AAAD. We also identified that the cell cycle pathway was significantly enriched by DEGs between normal and AAAD samples. Smooth muscle cells play important roles in maintaining vascular function and structural integrity. Dysfunctions of cell cycle in VSMCs are closely related to the aortic progressive dilation and ultimately rupture (21). Furthermore, we found that the ECM-receptor pathway was also significantly enriched by DEGs. The mechanical integrity of the aorta wall is determined by an ECM with appropriate structure. Elastin and collagen are crucial structural components of the ECM, which contribute to the stability and elasticity of normal arteries. ECM degradation also contributes to the progression of aortic dissection. Thus, we suggested cell cycle and ECM-receptor pathways might play important roles in AAAD.

Notably, upregulated CDC20, CDK1, CHEK1, KIF20A, MCM10, PBK, PTTG1, RACGAP, and TOP2A were hub nodes in the PPI network. Cell division cycle 20 homologue (CDC20) plays an important role in cell cycle progress by activating the APC E3 ubiquitin ligase, which initiates chromatid separation and entrance into anaphase (22,23). Checkpoint kinase 1 (CHEK1) plays an important role in the S and G2 cell cycle checkpoints. Inhibition of CHEK1 strongly increased replication stress and DNA damage, and this correlated with increased cell death (24). Pituitary tumor transforming gene 1 (PTTG1) has been shown to promote the expression of bFGF (25) and VEGF (26). bFGF induces cell proliferation and migration (27) and acts as an angiogenic factor that induces proliferation, migration, and differentiation of endothelial cells (28). VEGF is a multifunctional cytokine that acts as an effective chemokine, proliferator, and survival factor of endothelial cells (29). CDC20, CHEK1, and PTTG1 were found to be enriched in the cell cycle pathway and progress, and abnormalities in the pathway and progress may contribute to cell death. Although KIF20A, MCM10, PBK, and RACGAP were not enriched in the cell cycle pathway, their GO terms were enriched in biological processes associated with cell cycle, like cell division, mitotic cell cycle, and regulation of cell cycle. Moreover, these DEGs were proven to be hub genes in the PPI network, indicating that they might also play leading roles in DEGs. The DEGs mentioned above might be therapeutic targets for AAAD in the future.

Although cyclin-dependent kinase 1 (CDK1) was not enriched in any pathway and biological process in the present study, it was one of the hub nodes in the PPI network, indicating that CDK1 was also important in AAAD. This is supported by previous studies. CDK1 is a protein that regulates the cell cycle and belongs to the serine/threonine kinase family (30). Some studies have found that inhibition of CDK1 can decrease the proliferation and migration of VSMCs (31–34).

The miRNAs can regulate gene expression after transcription, and play key regulatory roles in AAAD. Various cell types including endothelial cells, smooth muscle cells (SMC), and immune cells in the vascular system play important roles in the development and progress of aortic dissection. Several miRNAs have been shown to target these cell types, especially vascular SMCs. In the present research, potential miRNAs of DEGs were identified. The miR-130/301 family controls collagen deposition and remodeling through PPARγ-APOE-LRP8 signaling. MiR-130/301 promotes vascular ECM remodeling and regulates vascular cell proliferation (35). MiR-302 family controls the phenotype of VSMCs and endothelial cells (ECs) via targeting type II BMP receptor (BMPRII) and type II receptor of TGFβs (TβRII) (36). Abnormality in these signaling pathways can lead to vascular disorders. Moreover, these microRNA families target DEGs that are involved in cell cycle progression. Therefore, we speculate that miR-130/301 family and miR-302 family might play important roles in AAAD.

Furthermore, the DEGs were analyzed in the CTD database, and several small molecules were predicted to correlate with AAAD. Palbociclib, a CDK4/6 inhibitor, was found to be effective in hormone receptor-positive breast cancer patients (37), while it is unknown if it has effects on AAAD. Dasatinib, a protein tyrosine kinase inhibitor, inhibited PDGF-stimulated migration and proliferation of human aortic SMCs (38). Azathioprine and zoledronic acid might be potential drugs for the prevention and treatment of AAAD. Azathioprine has beneficial effects on endothelial cells (ECs). Azathioprine increases ECs survival and maintains the contractile phenotype of SMC. Moreover, azathioprine reduces JNK activation and inflammation in the aortic vessel wall, and can inhibit aneurysm progression (39). Zoledronic acid can reduce Ang II-induced aneurysm formation and attenuate the expansion of the aorta. It can also attenuate elastin degradation, suppress MMP-2 activity, and decrease macrophage infiltration in the aortic tissues (40), while it is unknown whether azathioprine and zoledronic acid have effects on AAAD. Therefore, it is necessary to further study whether these two small molecules can be used in the treatment of AAAD.

There were some limitations to the present study. These results were obtained only through bioinformatics analysis, and they were not demonstrated by real-time polymerase chain reaction or animal models. Furthermore, although the DEGs, hub genes, potential miRNAs, and drugs were identified for AAAD, the study with bioinformatics analysis is just the first step and there is still a long way to translate these findings into clinical application. Despite these limitations, the results might provide new insights into the molecular mechanism, therapeutic targets, and potential drugs for AAAD.
